# Decoding position from multiunit activity using a marked point process filter

**DOI:** 10.1186/1471-2202-16-S1-P66

**Published:** 2015-12-18

**Authors:** Xinyi Deng, Daniel F Liu, Kenneth Kay, Loren M Frank, Uri T Eden

**Affiliations:** 1 Department of Mathematics and Statistics, Boston University, Boston, MA, USA; 2 UC Berkeley--UCSF Graduate Program in Bioengineering, UCSF, San Francisco, CA, USA; 3 Department of Physiology, UCSF, San Francisco, CA, USA

## 

Traditionally, experiments designed to study the role of specific spike patterns in learning and memory tasks take one of two forms, 1) observational studies that characterize statistical properties of neural activity during such tasks or 2) interventional studies that broadly alter neural activities over an entire neural population or brain region. This work is part of a larger project to allow investigators to manipulate neural populations in a content-specific way, altering spiking activity related to certain learning and memory patterns while leaving activity related to other patterns intact.

One fundamental challenge of this work is to decode the information content of specific spike sequences in real-time. Previously, we have used point process theory to develop efficient decoding algorithms based on spike train observations. However these algorithms assume the spike trains have been accurately sorted ahead of time, which is not possible for real-time decoding. Here we present a new point process decoding algorithm that does not require multiunit signals to be sorted. We use the theory of marked point processes to characterize the relationship between the coding properties of multiunit activity and features of the spike waveforms [1-3]. Using Bayes' rule, we compute the posterior distribution of a signal to decode given multiunit activity from a neural population.

We first characterize the spiking activity of a neural population using the conditional intensity function for marked point processes. We then construct point process filters to iteratively calculate the full posterior density of a signal. We illustrate our approach with a simulation study as well as with experimental data recorded in the hippocampus of a rat performing a spatial memory task. Our decoding framework is used to reconstruct the animal's position from unsorted multiunit spiking activity. We then compare the quality of fit of our decoding framework to that of a traditional spike-sorting and decoding framework. Our analyses show that the proposed decoding algorithm performs as well as or better than algorithms based on sorted single-unit activity. These results provide a mechanism for content-specific manipulations of population activity in hippocampus.

## References

[B1] Chen Z Kloosterman F Layton S Wilson MA Transductive Neural Decoding for Unsorted Neuronal Spikes of Rat Hippocampus Proceedings of the 34th Annual International Conference of the IEEE EMBS 2012 10.1109/EMBC.2012.6346178 PMC3972894 23366139

[B2] Cox DR Isham V Point Processes 1980 London: Chapman and Hall

[B3] Kloosterman F Layton S Chen Z Wilson MA Bayesian decoding using unsorted spikes in the rat hipposcampus J Neurophysiol 111 217 227 24089403 10.1152/jn.01046.2012 PMC3921373

